# Is later-life depression a risk factor for Alzheimer’s disease or a prodromal symptom: a study using post-mortem human brain tissue?

**DOI:** 10.1186/s13195-023-01299-2

**Published:** 2023-09-12

**Authors:** Lindsey I. Sinclair, Asher Mohr, Mizuki Morisaki, Martin Edmondson, Selina Chan, A. Bone-Connaughton, Gustavo Turecki, Seth Love

**Affiliations:** 1 Dementia Research Group, Faculty of Health Sciences, University of Bristol, Southmead Hospital, Level 1 Learning & Research Building, Bristol, BS10 5NB UK; 2 Douglas Institute, Department of Psychiatry, McGill University, Montreal, Canada; 3 Department of Life Sciences, Warwick University, Warwick, UK

**Keywords:** Depressive disorder, Alzheimer’s disease, Amyloid, Tau, Blood–brain barrier, Vascular depression, Dementia

## Abstract

**Background:**

Depression and dementia are both common diseases. Although new cases of depression are more common in younger adults, there is a second peak at the age of 50 years suggesting a different pathological process. Late-life depression (LLD) is associated with dementia. However, it remains unclear whether depression represents a dementia prodrome or is a true risk factor for its development.

LLD is thought to have a vascular component and this may be a possible link between depression and dementia. We hypothesised that later-life depression is a prodromal manifestation of dementia and would therefore be associated with more AD, and/or ischaemic brain abnormalities that are present in earlier-life depression or in age- and sex-matched controls.

**Methods:**

We assessed post-mortem orbitofrontal cortex and dorsolateral pre-frontal cortex from 145 individuals in 4 groups: 28 18–50-year-olds with depression, 30 older individuals (ages 51–90) with depression, 28 with early AD (Braak tangle stages III–IV) and 57 matched controls (17 early-life, 42 later-life). Levels of Aβ, phospho-tau and α-synuclein were assessed by immunohistochemistry and ELISA. To quantify chronic ischaemia, VEGF, MAG and PLP1 were measured by ELISA. To assess pericyte damage, PDGFRB was measured by ELISA. For blood–brain barrier leakiness, JAM-A, claudin 5 and fibrinogen were measured by ELISA. To quantity endothelial activation, the ratio of ICAM1:collagen IV was assessed by immunohistochemistry.

**Results:**

There was no evidence of chronic cerebral hypoperfusion or increased Aβ/tau in either depression group. There was also no indication of pericyte damage, increased blood–brain barrier leakiness or endothelial activation in the OFC or DLPFC in the depression groups.

**Conclusions:**

Contrary to some previous findings, we have not found evidence of impaired vascular function or increased Aβ in LLD. Our study had a relatively small sample size and limitations in the availability of clinical data. These results suggest that depression is a risk factor for dementia rather than an early manifestation of AD or a consequence of cerebral vascular insufficiency.

**Supplementary Information:**

The online version contains supplementary material available at 10.1186/s13195-023-01299-2.

## Background

Depression and Alzheimer’s disease (AD) are both common diseases. Biological, psychological and social factors are known to be involved in the predisposition, precipitation and perpetuation of depressive episodes. In this study, we focused on biological factors. Although new cases of depression are more common in younger adults, there is a second peak in the 50 s suggesting a different pathological process [1]. The phenomenology of depression in later life differs from that earlier in life, with somatic symptoms being more prominent [2]. Approximately 1/3 of patients with depression are resistant to treatment [3]. Alzheimer’s disease (AD) is the most common form of late-life dementia. Its incidence is set to nearly triple by 2050, due to the ageing of the population [4].

Cognitive symptoms may occur in depression [5], particularly in the elderly in whom depressive ‘pseudodementia’ has long been recognised, although this term is no longer used to describe cognitive symptoms occurring in a depressive illness that seem to mimic dementia [6]. Although cognitive symptoms often improve with treatment of the depressive illness, there is some evidence that the risk of subsequent dementia is raised [7]. Cognitive dysfunction can persist even after the episode of depression [8–10], particularly for those with persistent depressive symptoms [11, 12]. It has been shown that hippocampal volume is reduced in individuals with depression [13, 14].

Building on previous research (e.g. [12, 15]), depression was identified by the Lancet Commission in 2017 as a potential modifiable risk factor for the development of AD [16]. Whilst some studies support this view, others suggest that, rather than being a risk factor for AD, later-life depression reflects prodromal symptoms of AD [17–21]. Several possible mechanisms for this link have been suggested, e.g. [12] including glucocorticoid excess, hippocampal atrophy, cognitive reserve and cerebrovascular dysfunction. It has been suggested that depression is associated with a milieu in the brain which lowers cognitive reserve via multiple mechanisms, thus leading to observed increased risk in dementia [12]. Post-mortem studies have attempted to resolve this question. Some studies have found more prominent AD pathology in those with a history of low mood, whilst others have not. Most of these studies have been relatively small and looked at milder depression [22–24]. Neuroimaging studies have reported cortical thinning in those with a history of depression [25, 26]. It remains unclear what the exact relationship is between depression and AD. Depression is also common in Lewy body dementia [27].

Vascular risk factors such as hypertension and diabetes are common in middle age, affecting over ¼ of individuals in some studies [11]. The ‘vascular depression’ hypothesis of later-life depression was first suggested in 1997 [28]. It is supported by both imaging and epidemiological evidence, e.g. [29–31]. The direction and nature of the relationship between the vascular changes and depression remains unclear. Most previous studies have focused on focal vascular pathology, e.g. infarcts, and have not found any link [32]. The authors proposed that depression and vascular disease may have a common underlying biological substrate. There is evidence from diffusion tensor imaging studies that both depression and disturbances of cognition are associated with diffuse white matter damage, which is difficult to detect and quantify by conventional histological methods [33, 34].

It is increasingly recognised that vascular dysfunction plays a major role in the development of AD. Many of the genes that influence the risk of AD are highly expressed by cells in the cerebral vasculature [35]. It is currently thought that cerebrovascular disease both increases the risk of AD developing and accelerates its progression and that non-structural abnormalities such as blood–brain barrier dysfunction may be involved [36]. It is therefore plausible that vascular disease may underlie the link between later-life depression and AD.

We aimed to contribute additional evidence to better understand the complex relationship between depression and AD. Specifically, we have studied post-mortem tissue from brain areas known to be affected by depression and/or AD to determine whether individuals with depression have increased core AD neuropathology, evidence of chronic hypoperfusion, increased blood–brain barrier leakiness or pericyte damage. We chose to study the medial temporal cortex, as it is one of the earliest brain regions affected in Alzheimer’s disease, and differences in brain structure in the medial temporal lobe have been demonstrated in depression [37, 38]. We chose the orbitofrontal cortex (OFC) as volume loss (grey matter) has been demonstrated in the OFC in depression, which can be reversed with antidepressants [39, 40]. Amyloid beta is found in the OFC early in the course of AD and it is thought to be part of the core hub of the Aβ network [37, 41]. The dorsolateral prefrontal cortex (DLPFC) has been robustly shown to demonstrate hypometabolism in depression [42] and severity of depression has been shown to negatively correlate with grey matter volume in the DLPFC [43]. The DLPFC is also affected in Alzheimer’s disease, but later in the disease process [37].

## Methods

Ethical approval for this work was provided by the Greater Manchester East NRES committee (ref 17/NW/0126). Tissue was supplied by the Douglas Bell Brain Bank from individuals with depression, early AD and controls. Tissue was supplied by the South West Dementia Brain Bank (SWDBB) from individuals with early AD and older controls. As far as practicable, cases and controls were matched for age, gender and post-mortem interval. Comprehensive clinical summaries are recorded in the SWDBB database for all brain donors. The summaries are generated from the patient medical notes and from any additional information collected as part of the Brains for Dementia Research Programme. In addition to information from clinical notes, the Douglas Bell Brain Bank has a long-standing arrangement with the Quebec coroner’s office which allows collection of brain tissue from individuals who have died by suicide, with next-of-kin consent. Approximately 4 months after donation, a trained researcher visits relatives, with permission, to perform a psychological autopsy with 2 different informants. The combining of data from more than a single source means that diagnostic information is more detailed and reliable.

### Case selection

For the depression groups, individuals had to have had depression meeting DSM-IV or DSM-V criteria (depending on the date of donation) either at the time of their death or in the 2 years preceding their demise. Individuals were excluded if they had evidence of a dementing illness either ante-mortem or on post-mortem examination, or major neuropathological abnormalities post-mortem affecting the brain areas under investigation (e.g. cancer) or evidence of a widespread neurological disease process other than those under investigation (e.g. multiple sclerosis). Individuals aged < 50 at death were classed as having early-life depression and those > 50 were classed as having later-life depression. This definition is by its very nature somewhat arbitrary and there has been considerable debate about the age at which middle age is thought to start. We chose this cut-off as an age where half of the population would have gone through menopause [44] which is a well -recognised transition point to middle age.

In the early AD group, individuals had to have had a diagnosis of MCI/AD made in the 2 years before death and a Braak tangle stage of IV or less. They were excluded if they had a history of a major depressive disorder severe enough to require psychiatric treatment or if they had a major neuropathological abnormality at post-mortem affecting the brain areas under investigation (e.g. cancer) or evidence of a widespread disease process other than those under investigation (e.g. multiple sclerosis).

For the control groups, individuals were required to have no history of a major depressive disorder severe enough to require psychiatric treatment and no evidence of a dementing illness either ante-mortem or at post-mortem examination. They were excluded if they had a major neuropathological abnormality at post-mortem affecting the brain areas under investigation (e.g. cancer) or evidence of a widespread disease process other than those under investigation (e.g. multiple sclerosis).

### Homogenate preparation

Fresh frozen tissue was dissected from the occipital frontal cortex and dorsolateral prefrontal cortex. To produce the SDS homogenates used for most of the assays in this project, 200 mg of tissue was homogenised in 1 ml of chilled 1% SDS lysis buffer in a Precellys tissue homogeniser (2 × 15 s at 6000 g) with 6–10 zirconia beads in a 2-ml homogenate tube. The homogenates were then centrifuged for 15 min at 13,000 g at 4 °C. Supernatant was aliquoted into non-binding 96-well storage plates (Thermo Scientific) and frozen at − 80 °C until required. The soluble and insoluble fraction homogenates used for the amyloid beta assays were produced in a two-step process. Firstly, 200 mg of tissue was homogenised in 1 ml of chilled TBS with 1% NP-40 lysis buffer in a Precellys tissue homogeniser (2 × 15 s at 6000 g) with 6–10 zirconia beads in a 2-ml homogenate tube. The homogenates were then centrifuged for 15 min at 13,000 g at 4 °C. The supernatant (the soluble fraction) was removed, aliquoted into non-binding 96-well storage plates (Thermo Scientific) and frozen at − 80 °C until required. The insoluble fraction was obtained by adding 400 μl of guanidine extraction buffer (3 parts guanidine-HCl to 1 part 50 mM Tris pH 8) and homogenising in a Precellys tissue homogeniser (2 × 15 s at 6000 g). The samples were then incubated for 4 h at 25 °C before being centrifuged for 15 min at 13,000 g at 4 °C. The supernatant (the insoluble fraction) was removed, aliquoted into non-binding 96-well storage plates (Thermo Scientific) and frozen at − 80 °C until required.

### Preparation of fixed sections and immunohistochemistry

Formalin-fixed tissue was fixed for 3–4 weeks in formalin prior to being dissected into blocks and embedded in wax. Paraffin sections of the orbitofrontal cortex, dorsolateral prefrontal cortex, and medial temporal lobe were cut at 7-μM thickness. Immunolabelling for amyloid beta and tau was performed in an automated immunostainer (Ventana BenchMark ULTRA, Roche Tissue Diagnostics). Amyloid beta was labelled using the pan Aβ 4G8 antibody (Millipore) diluted 1:8000 in PBS. Tau was labelled using the AT8 phospho-tau antibody diluted 1:500 in PBS (Fujirebio).

Sections for collagen IV were dewaxed (2 × 5 min in clearene), blocked in 3% hydrogen peroxide in methanol for 30 min and boiled in citrate buffer for 15 min. Using a vectastain ABC elite kit (Vector laboratories), sections were blocked for 20 min prior to incubation overnight at rtp with the antibody (Sigma Aldrich C1926) diluted 1:500 in PBS. The next day sections were washed in PBS, incubated for 20 min with universal secondary antibody, washed again in PBS, incubated with the ABC reagent for 20 min, washed in PBS and incubated with DAB for 10 min before being washed in running water for 10 min. Finally, sections were immersed in copper sulphate solution for 4 min before being washed again in running water for 5 min, counterstained in haemotoxylin for 20 s, washed again in running water for 15 min before dehydration in 100% alcohol (2 × 2 min) and clearing in clearene (2 × 5 min) prior to mounting. Sections for ICAM-1 were stained as for collagen IV except that the antibody used (BBA17 R&D systems) was diluted 1:300 in PBS and the sections were only boiled in citrate buffer for 10 min.

The field fraction immunopositive for each antigen was assessed by examining up to 15 random fields from each brain area under a × 20 objective. The software package Image Pro Plus 7 (Media Cybernetics, MD, USA) was used to select the fields at random in the pre-defined area and for image capture. For a small number of slides, the area of cortex included in the section was too small to accommodate 15 non-overlapping fields but at least 10 were captured in all cases.

### ELISAs

VEGF, MAG, PLP1 and alpha-synuclein were measured by ELISA as previously described in detail [45]. We have previously demonstrated that MAG, PLP, vWF, soluble and insoluble Aβ and VEGF are stable for up to 72 h post-mortem [46–49]. vWF was measured by dot blot as previously described in detail [45]. Aβ 40 and 42 were assayed using a kit-based ELISA (R&D systems, DAB142 and DAB140B). Samples were diluted 1:2000 except for samples with a Braak stage ≥ IV or any samples where the amount of amyloid was greater than the top standard when diluted at 1:2000. These samples were diluted 1:10,000. A small number of samples required dilution at 1:20,000 to achieve a result which could be interpolated. JAM-A was assayed using a kit-based ELISA (BosterBio EK1349) as per the manufacturer’s instructions. Samples (NP-40 soluble fraction) were diluted 1:5.4 in the diluent supplied. Haemoglobin was measured using a kit (Cayman Chemical, CAY700540), as per the manufacturer’s instructions, with 20 μl of sample added to 180 μl in each well. PDGFRβ was assayed using a kit-based ELISA (R&D systems DYC385-5). Samples were diluted 1:60 in PBS. All samples in all assays were measured in duplicate and at least 2 carry-over samples were used on each plate to ensure cross-plate consistency.

Occludin was measured in the soluble fraction by sandwich ELISA in 96-well nunc maxisorp plates. The plate was coated with the capture antibody (ab31721 Abcam, Cambridge) diluted 1:1000 in PBS and incubated overnight at 4 °C. The next day the plate was washed × 5 in PBS/5% tween and then blocked with 300 μl of 1% BSA/PBS per well for 2 h at 25 °C at 500 rpm. After a further 5 washes in PBS/tween, the samples were diluted (OFC 1:25, DLPFC 1:30) in 1% BSA/PBS and 100 μl was loaded in duplicate for each sample. Blanks consisted of 100 μl 1%BSA/PBS. The top standard was diluted in 1% BSA/PBS to a concentration of 428.6 ng/ml with 6 further twofold dilutions (H00004950-P101, Abnova) and 100 μl was loaded in duplicate for each dilution. All samples were incubated for 2 h at 25 °C at 500 rpm. Following 5 washes in PBS/tween, 100 μl of the detection antibody (OC-3F10, ThermoFisher Scientific) diluted 1:2000 in PBS was loaded per well and incubated for 2 h at 25 °C at 500 rpm. After a final 5 washes in PBS/tween, 100 μl of the colorimetric reagent (R&D systems) was added to each well. After 20 min, 50 μl of STOP solution was added to each well. Absorbance was read at 450 nm.

Claudin 5 was measured in the soluble fraction by sandwich ELISA in 96-well nunc maxisorp plates. The plate was coated with the capture antibody (4C3C2, ThermoFisher Scientific) diluted 1:1000 in PBS and incubated overnight at room temperature. The next day the plate was washed × 5 in PBS/5% tween and then blocked with 300 μl of 1% BSA/PBS per well for 2 h at 25 °C at 500 rpm. After a further 5 washes in PBS/tween, the samples were diluted 1:100 in 1% BSA/PBS and 100 μl was loaded in duplicate for each sample. Blanks consisted of 100 μl 1%BSA/PBS. The top standard was diluted in 1% BSA/PBS to a concentration of 167 ng/ml with 6 further twofold dilutions (Abnova) and 100 μl was loaded in duplicate for each dilution. All samples were incubated overnight at 4 °C. Following 5 washes in PBS/tween, 100 μl of the detection antibody (ab131259, Abcam) diluted 1:4000 in 1% BSA/PBS was loaded per well and incubated for 2 h at 25 °C at 500 rpm. After 5 further washes in PBS/tween, 100 μl of goat anti-rabbit HRP conjugated antibody (Vector laboratories) was added to each well and incubated for 40 min at 25 °C at 500 rpm in the dark. After a final 5 washes in PBS/tween, 100 μl of the colorimetric reagent (R&D systems) was added to each well. After 20 min, 50 μl of STOP solution was added to each well. Absorbance was read at 450 nm.

### Statistical analysis

All ELISAs were adjusted for total protein content. This was done using the formula

$$\mathrm{Adjusted\,result\,}=\frac{\mathrm{sample\,target\,protein\,concentration}}{\mathrm{sample\,total\,protein\,concentration}}$$

All data were analysed using parametric statistical tests such as ANOVA wherever possible. If a variable was not normally distributed and it was not possible to achieve a normal distribution by transformation, non-parametric tests were used such as the Kruskal–Wallis test. The primary outcome measure was whether Aβ measured by immunohistochemistry (the current gold standard) varied between groups. For field fraction analysis, each field on each slide was given equal weighting, and mean values were compared by ANOVA, with age, gender and post-mortem interval as co-variates. For a priori analyses, any demographic variable which varied between groups (e.g. age, post-mortem interval) was included as a covariate. Where Dunn’s test was used, Bonferroni correction was applied. A threshold *p* value of 0.05 was used throughout.

As we had relevant pilot data, no formal power calculation was possible, but we estimate using information from previous studies that we had > 90% power to detect a difference of 2% between groups in the field fraction positive for Aβ and 83% power to detect a difference of 0.6 between groups in the MAG:PLP1 ratio [45, 50].

## Results

Tissue was available from 145 individuals: 28 with early-life depression, 30 with later-life depression, 28 with early AD, 17 early-life controls, 23 later-life controls and 19 AD controls (Table 1). As would be expected, there were clear differences in age between the groups. There was no statistical evidence of a difference in gender between the groups. The later-life depression and later-life controls had longer PM delays. All of the individuals in the depression groups had died by suicide and all had a DSM axis 1 diagnosis of either major depressive disorder or depressive disorder not otherwise specified. As shown by the cause of death and the medications used in the last 3 months, these were severely depressed individuals.

**Table 1:** Clinical and demographic information for the 145 individuals who donated tissue used in this study

	**Early life MDD (*n* = 28)**		**Later life MDD (*n* = 30)**		**Early life controls (*n* = 17)**		**Later life controls (*n* = 23)**		**AD controls (*n* = 19)**		**Early AD (*n* = 28)**		**Statistical evidence**
	Mean	SD	Mean	SD	Mean	SD	Mean	SD	Mean	SD	Mean	SD	
Age (y)	35.4	9.2	64.5	11.6	31.9	11.5	65.1	10.4	84.0	6.4​	83.714	6.1​	ANOVA *p* < 0.001
Gender													
*Male*	19		19		16		14		18		11		Chi^2^ test​, *p*=0.218
*Female*	9		11		1		9		10		8		
Pm delay (h)	48.3	18.3	57.6	27.1	34.1	20.0	56.9	28.0	55.3	22.4	42.9	29.5	Kruskal–Wallis *p* = 0.01
Cause of death													
*Suicide*	28		30		0		0		0		0		Chi^2^ test​, *p* < 0.001
*Accident*	0		0		8		4		5		0		
*Natural causes*	0		0		9		15		4		28		
DSM Axis 1 Diagnosis													
*Nil*	0		0		17		19		9		0		Chi^2^ test​, *p* < 0.001
*Major depressive disorder*	22		23		0		0		0		0		
*Depressive Disorder NOS*	6		7		0		0		0		0		
*Dementia*	0		0		0		0		0		28		
DSM Axis 1 dependence													
*Nil*	21		27		17		19		9		28		Chi^2^ test​, *p* = 0.158
*Substance*	6		3		0		0		0		0		
*Alcohol*	1		0		0		0		0		0		
Rx in last 3/12													
*Nil*	15		9		17		14		6		0		Chi^2^ test​, *p* < 0.001
*Antidepressant*	0		6		0		2		0		0		
*Antipsychotic*	0		0		0		0		1		0		
*Benzodiazepine*	1		4		0		0		1		1		
*Mood stabiliser*	1		1		0		0		0		0		
*Antidepressant* + *another class*	11		10		0		0		0		0		
*Unknown*	0		0		0		1		1		27		
Braak stage *(note high levels of missing data except in early AD group)*							0.25	0.50	2.3	0.7	3.773	0.43	Chi^2^ test​ *p* < 0.001

### Amyloid, tau and α-synuclein

**Fig. 1:**
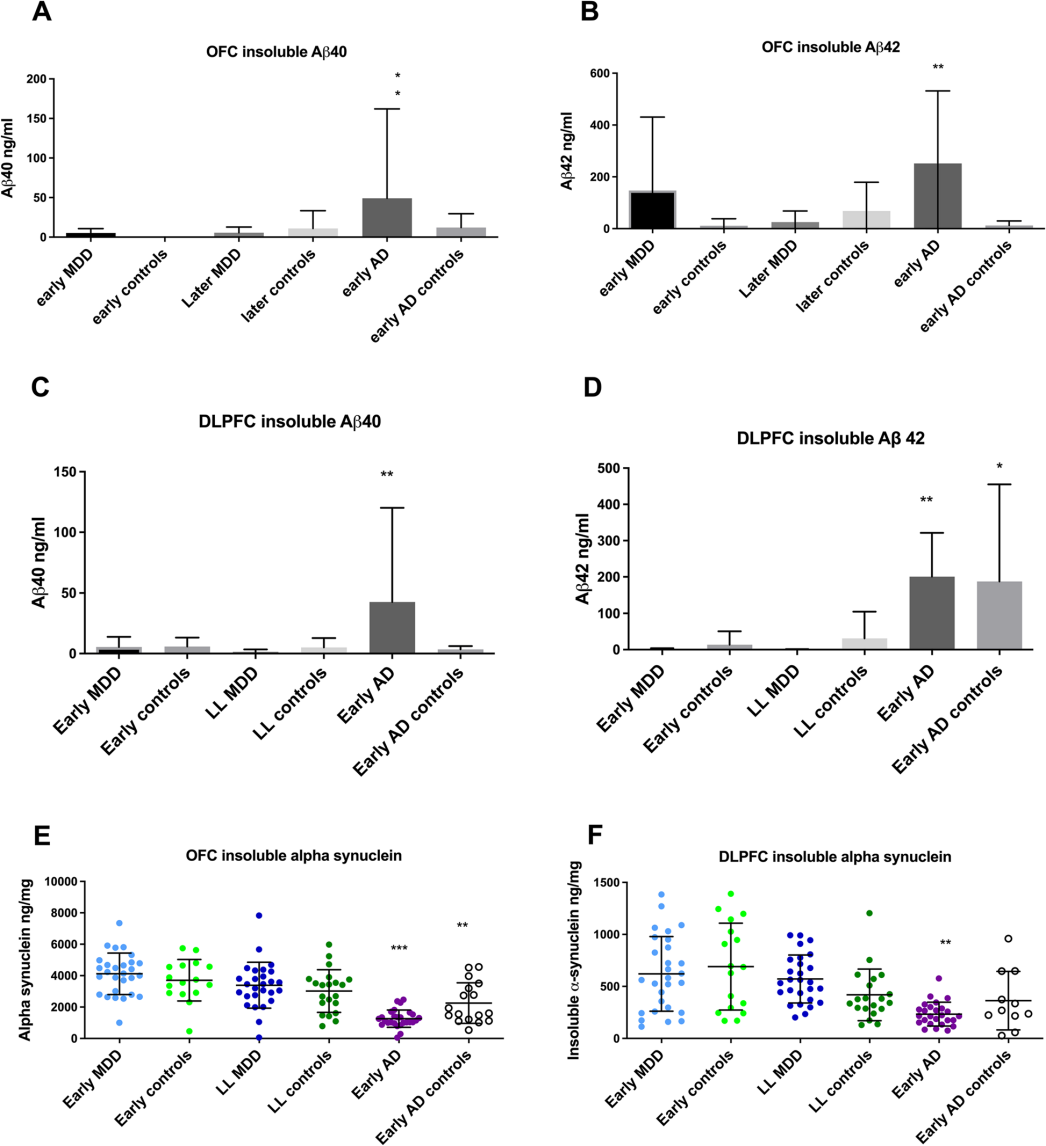
Markers of neurodegeneration. Insoluble Aβ 40 was increased in the early AD group in both the OFC (**A**) and DLPFC (**C**). Insoluble Aβ 42 was also increased in the early AD group in the OFC (**B**) and DLPFC (**D**). Conversely, insoluble α-synuclein was decreased in the early AD groups in the OFC (**E**) and DLPFC (**F**). In the DLPFC, insoluble Aβ 42 (**D**) was increased in both the early AD and early AD control groups compared to all other groups. There was no difference between the early AD and early AD control groups in insoluble Aβ 42

As shown in Fig. 1, insoluble amyloid β 40 and 42 were elevated in both the OFC and DLPFC in the early AD group. Insoluble Aβ 42 was also increased in the DLPFC in the early AD control group compared to all other groups except early AD. There was no evidence of an increase in insoluble amyloid β 40 and 42 in the later-life depression group. Insoluble α-synuclein was decreased in the early AD group in both the OFC and DLPFC. Again there was no evidence of increased α-synuclein in the later-life depression group. This suggests that later-life depression is not due to incipient Lewy body or Aβ-related neurodegeneration.

**Fig. 2:**
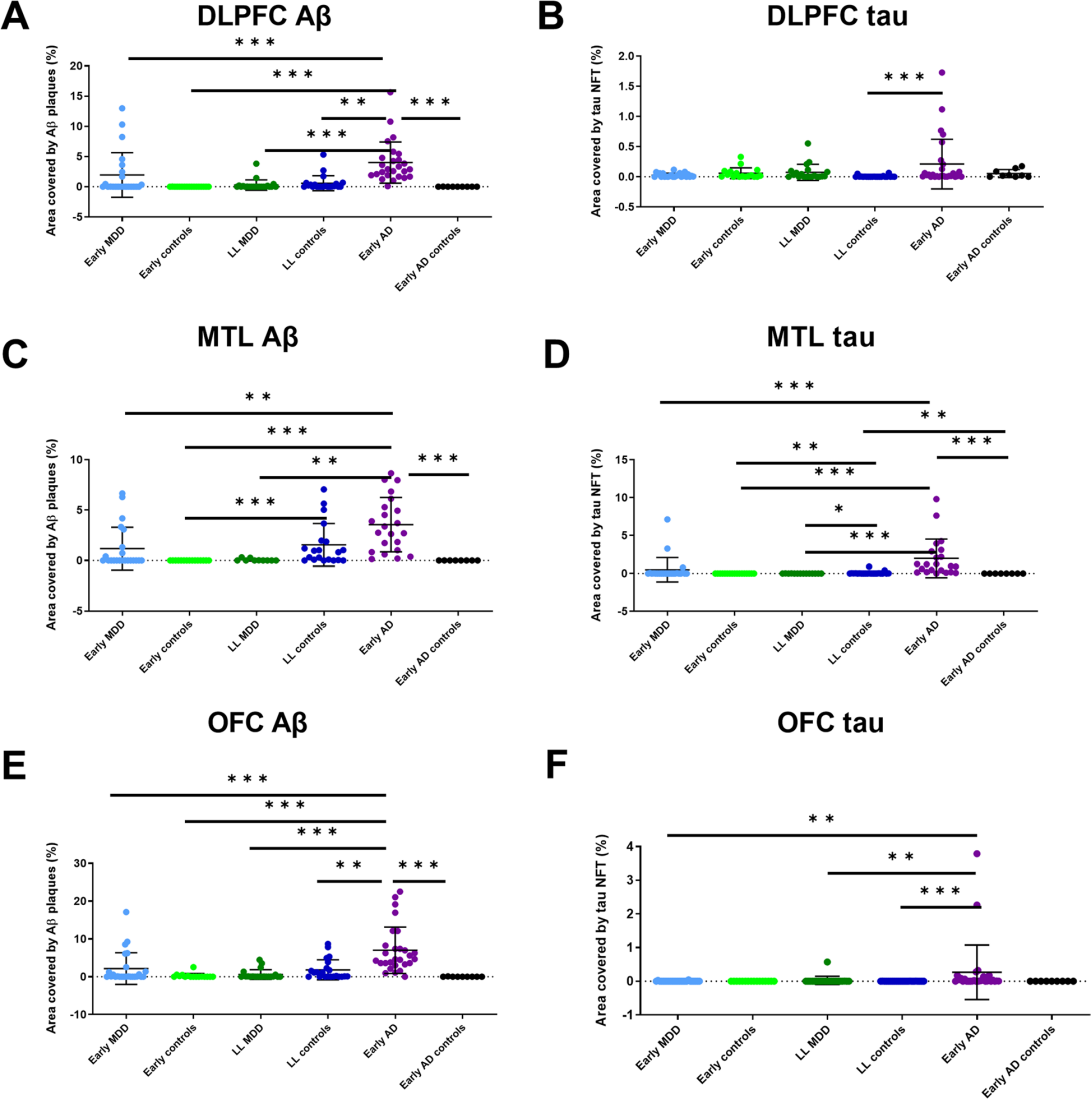
Aβ and tau loads measured by immunohistochemistry and field fraction analysis in the DLPFC (**A**, **B**), medial temporal lobe (**C**, **D**) and orbitofrontal cortex (**E**, **F**). In all areas, the expected increase of both proteins was seen in the early AD group compared to controls

Assessment of Aβ and tau by immunohistochemistry (see Fig. 2) showed, as expected, that both were increased in the early AD group in the medial temporal lobe, OFC and DLPFC. In the medial temporal lobe, Aβ load was higher in the early AD group than in early AD control (*X*^2^ = 57.52, *p* < 0.001) and tau load was higher in later-life controls than in the later-life depression group (*X*^2^ = 31.76, *p* = 0.046). This again suggests that later-life depression is not a result of neurodegeneration.

### Measures of chronic hypoperfusion

**Fig. 3:**
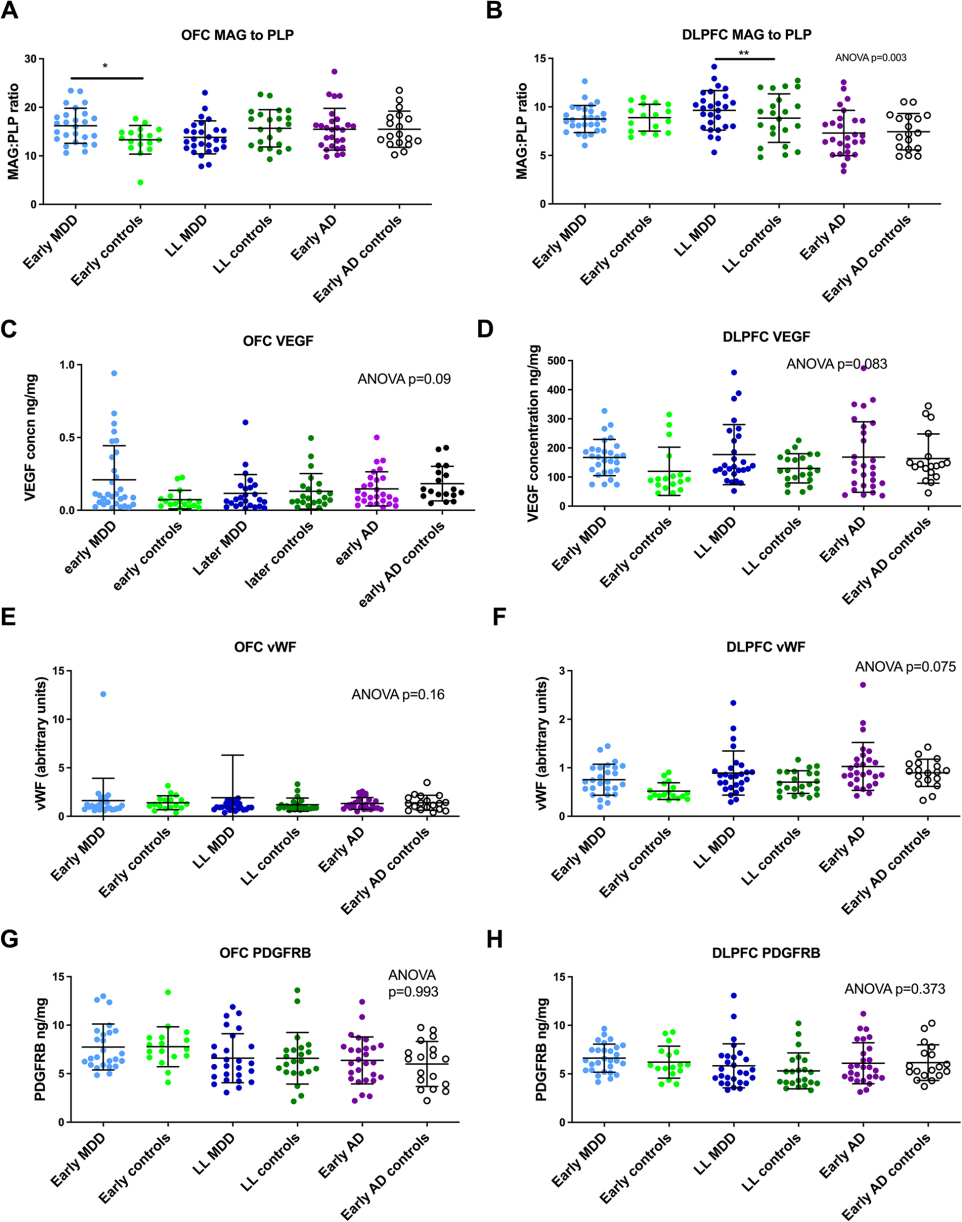
Markers of perfusion and angiogenesis. The expected reduction in MAG:PLP ratio was not seen in either the OFC (**A**) or DLPFC (**B**). Neither was there evidence of increased angiogenesis (**C**, **D**) or microvessel density (**E**, **F**) or pericyte damage (**G**, **H**)

If chronic hypoperfusion had been present, MAG would have been reduced compared to PLP1. There was no evidence of this in either the early- or later-life depression groups (see Fig. 3). Reduction in MAG:PLP1 ratio was not seen in the early AD group, suggesting that this may occur later in the disease process in the OFC and DLPFC. There was no evidence of a between-group difference in vWF or VEGF as would have been expected had there been a difference in either microvessel density or angiogenesis. There was no difference in the pericyte marker PDGFRβ. Overall therefore, we found no evidence of a between-group difference in markers of chronic perfusion or vessel damage.

### Blood–brain barrier

**Fig. 4:**
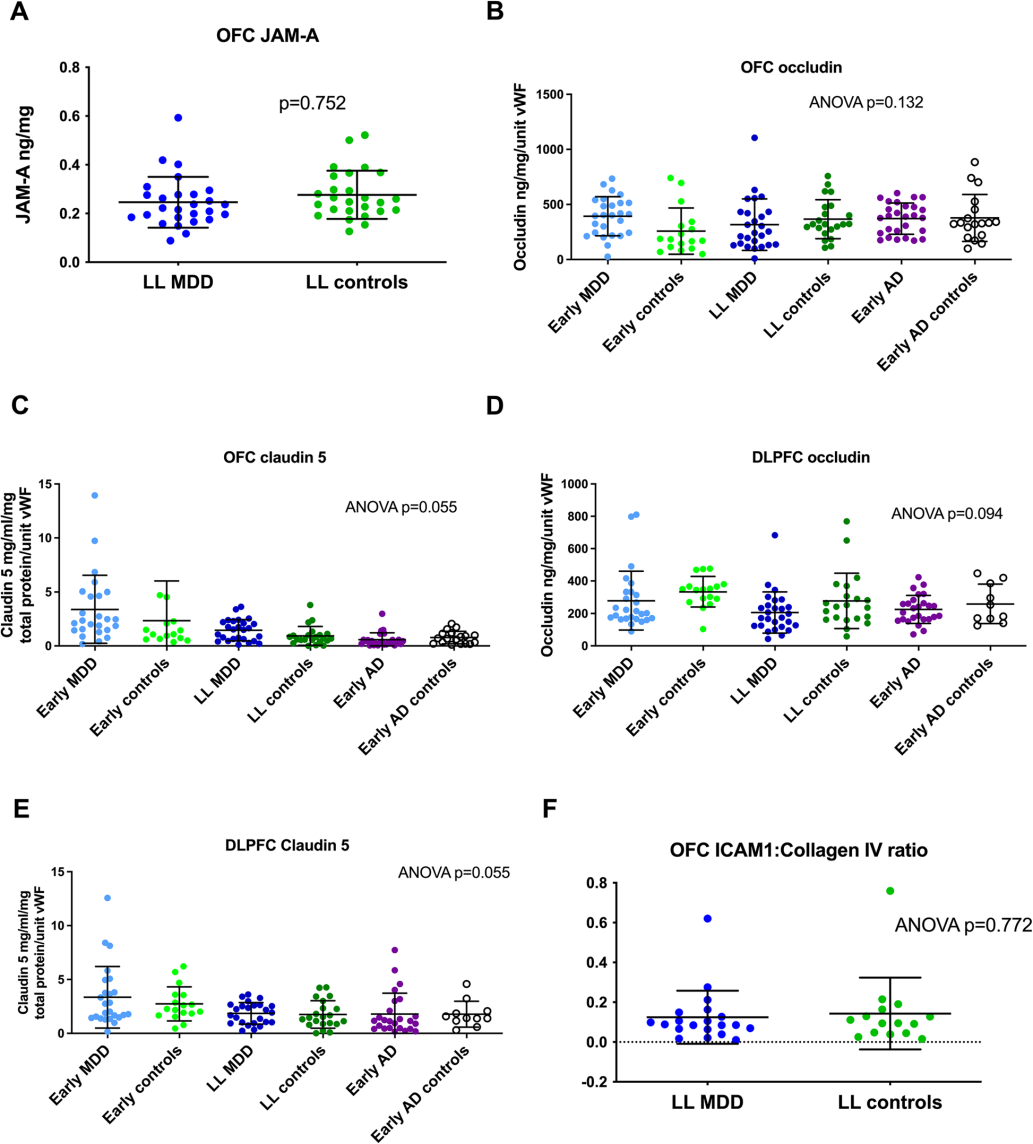
Blood–brain barrier integrity and endothelial activation. There was no evidence of a between-group difference in JAM-A (**A**), occludin (**B**, **D**), claudin-5 (**C**, **E**) or ICAM-1:collagen ratio (**F**)

Finally, we studied the blood–brain barrier to measure markers of leakiness and endothelial activation. We measured three tight cell junction proteins (JAM-A, claudin 5 and occludin), and additionally the ICAM-1/collagen IV ratio in the later-life depression and later-life control groups. There was no evidence of a reduction in any of the tight cell junction proteins in the later-life depression group (see Fig. 4). Likewise, the ICAM-1:collagen-IV ratio was unchanged in the later-life depression group compared to controls in the OFC. Although there were differences in the DLPFC (Supplementary Figure 1), these were attributable to a single outlier value and the absence of labelling of ICAM-1 in the majority of sections. Together, these results provide strong evidence that later-life depression is not associated with blood–brain barrier leakiness or endothelial activation.

## Discussion

In summary, we have found, using a mixture of ELISAs and immunohistochemistry, no evidence that later-life depression is associated with an increase in insoluble Aβ or α-synuclein in the OFC or DLPFC, or increased immunolabelling of Aβ or hyperphosphorylated tau in the OFC, DLPFC or medial temporal lobe. Later-life depression was not associated with changes in markers of chronic hypoperfusion or angiogenesis—MAG:PLP1, vWF and VEGF—that we previously demonstrated to be abnormal in AD. Finally, we found no evidence of increased blood–brain barrier leakiness in later-life depression. At least in our cohort, therefore later-life depression does not appear to have been vascular in origin, or the result of early AD- or Lewy body-type neurodegenerative change.

Later-life depression can be influenced by life events and psychological factors, as well as by biological differences. For example in later life, losses are common, e.g. bereavement, loss of role as well as co-morbid physical health conditions. In this study, we were not able to examine the influence of these factors. Previous studies that examined depression in close proximity to the development of cognitive problems, or depression some years before cognitive decline, have yielded conflicting results. Some found a relationship between amyloid and later-life depression, whereas others, like ours, did not. It has been suggested that later-life depression is probably heterogeneous and that this may explain some of the discrepant findings. Another explanation, outlined in [12], is that depression acts via multiple mechanisms to reduce cognitive reserve, thus making dementia more likely. It is also possible that later-life depression is an early sign of dementia in some people, but not in others as well as being a risk factor for the development of dementia [12, 15].

In one of the earliest amyloid PET studies (*n* = 39), Kumar et al. found, in a sample recruited via advertisement, that individuals in their mid-60 s with later-life depression had higher amyloid binding in multiple brain regions [51]. This finding was partly replicated by Wu et al. in a similar-sized and aged cohort, but they found that amyloid binding in those with a lifetime history of depression was increased in the precuneus and parietal lobe only [52].

In a moderately sized study (*n* = 145) of people with later-life depression (mean age 72) with and without evidence of AD, in addition to healthy controls and those with AD, those with later-life depression even without evidence of AD had decreased CSF Aβ42:40 ratios and performed poorly on memory tests [53]. Donovan et al. reported that those with a history of depression (*n*–35) had higher amyloid binding at baseline and that higher baseline amyloid binding in the cohort as a whole was associated with worsening GDS scores over time. This suggested that depressive symptoms are part of an early constellation of AD symptoms [54]. Almdahl et al., using data from ADNI participants who were cognitively normal, found that those with incident depression during follow-up showed reduction in hippocampal volume (but only after onset), that higher white matter hyperintensity burden at baseline was associated with increased risk of incident depression, and that higher amyloid burden was present on PET at baseline, at depression onset and at the last visit in the depressed group [55].

Conversely Wilson et al., in the longitudinal ROS and MAP studies which used CES-D to monitor depressive symptoms, found that neither amyloid nor tau load measured by immunohistochemistry in 8 areas of brain from 582 individuals who had donated their brains (2/3 of whom had developed MCI or dementia) was related to depressive symptoms during follow-up [56]. This was replicated by McCutcheon et al. using NACC data, from individuals who were mostly in their mid-80 s. The authors demonstrated that in those with mild AD, normal cognition and MCI, there was no relationship between AD neuropathology and depressive symptoms at the last visit before death [57]. Their study differed from one by Rapp et al. which used a clinician diagnosis of depression rather than the GDS, and considered depression at baseline [24]. In a smaller imaging study (*n* = 68), only 7 of the later-life depression participants had an amyloid PET scan with a SUVR above the positive threshold [58]. In a moderate-sized PET study (*n* = 100) with participants in their early to mid-70 s, De Winter et al. found no evidence that those with later-life depression had higher amyloid binding or more white matter lesions [59]. In the Swedish BIOFINDER study, a longitudinal cohort with a mean age of 73.8 at baseline, there was no relationship between CSF AD biomarkers and depressive symptoms [60]. Mackin et al., using ADNI data (*n* = 238), although those with depression were recruited separately, identified individuals with late-life depression (1/3 of whom met criteria for MCI) and found that they had less cortical amyloid deposition than did non-depressed controls; furthermore, amyloid deposition was not associated with the number or severity of lifetime depressive episodes [61].

The theory that depression in later life may have a vascular origin was first posited in 1997 and many studies since have examined the link between depression and vascular damage [62]. The majority have looked at either radiological evidence of vascular changes, e.g. white matter hyperintensities (WMH), or studied the relationship between vascular risk factors and depression. Not all studies have shown an effect of depression but meta-analytic evidence has been more convincing [63]. In an extensive literature review, Taylor et al. argued that later-life depression had been consistently demonstrated to be related to greater WMH severity and volume, particularly in the DLPFC [62]. They acknowledged however that several previous neuropathological studies, like ours, had found no relationship between later-life depression and vascular dysfunction or cerebrovascular disease [22, 64, 65]. Those previous studies predominantly used immunohistochemical or macroscopic techniques to quantify cerebrovascular disease. Our study was able to measure aspects of microvascular dysfunction such as chronic hypoperfusion and blood–brain barrier leakiness that are difficult to assess morphologically.

It has been previously suggested that the relationship between depression and vascular dysfunction may be bidirectional [66]. For example, depression is known to be associated with a more sedentary lifestyle and poorer diet, possibly due to apathy [67, 68]. This could theoretically lead to an increased risk of atherosclerosis and vascular dysfunction. It has also been suggested, as discussed above, that depression itself may lead to vascular dysfunction but we found no evidence of this in our study.

In contrast to our findings, other authors have found evidence suggesting that depression in later life may lead to vascular dysfunction. For example, two recent analyses from the AGES-Reyjavik study examined the relationship between depression, cerebrovascular disease and dementia. In the first, the authors produced trajectories of GDS scores between baseline and follow-up at ~ 9 years [69]. They found that those with the most severe depression (i.e. steepest GDS trajectory) were more likely to develop dementia (HR = 1.81; 95% CI: 1.15–2.83; *p* = 0.01) and that this group had the greatest increase in white matter hyperintensities over the 9 years (42%, *p* = 0.03) versus those with no depression. The second analysis focused on modelling the relationship between depression and dementia. When white matter hyperintensities (WMH) were included in the model, the relationship between incident depression and dementia was attenuated but remained statistically significant [70]. It appeared that those with current depression in their study may have had more WMH than those with past WMH, but the authors did not report any statistical evidence for this. Kirton et al. found in a relatively small cohort (*n* = 116) that higher depressive symptoms were predictive of greater increases in WMH over time only in women and particularly in those who were older at baseline [71]. These studies did however almost all rely on MRI markers of white matter change as evidence of vascular dysfunction.

Far fewer studies have measured markers of vascular dysfunction in human post-mortem brain tissue. One of the few tissue-based studies that examined vWF in depression suggested that it was increased in older adults with depression, but this was a very small study (*n* = 25) [72]. In a very small gene expression study using PCR, no effect of depression was seen on the expression of either MAG or PLP1 [73]. Another similar-sized study found that MAG was decreased in depression [74].

Depression in later life has been reported to confer a greater risk of later vascular dementia than AD [75]. In a large meta-analysis of 38 studies examining carotid intima media thickness (CIMT), Wu et al. found that CIMT was slightly increased in individuals with depressive symptoms and that endothelial function was also reduced [52]. The effect on CIMT seemed mainly to be driven by studies that included older adults. In another large meta-analysis (*n* = 48 studies), van Agtmaal et al. analysed markers of microvascular dysfunction. They found no clear evidence of an effect of depression on plasma vWF (3 studies), but reported more cross-sectional white matter hyperintensities in those with depression (OR per standard deviation 1.29, *p* < 0.001) and an effect of white matter hyperintensities on incident depression in the 8 longitudinal studies examined (pooled OR 1.18, *p* < 0.001) [76]. These effects persisted after adjustment for cardiometabolic risk factors such as hypertension and diabetes. In a meta-analysis of 16 studies measuring plasma VEGF in individuals with depression versus controls, Tseng et al. found that plasma VEGF was elevated in individuals with depression, but few of the studies included older adults. It appeared from a secondary analysis that the effect declined with age [77]. In contrast, we have not found evidence that later-life depression is associated with cerebral hypoperfusion or other vascular dysfunction.

Endothelial cells in the CNS vasculature and the junctions between them form the blood–brain barrier (BBB) as shown in Fig. 2. The function of the BBB is to protect the brain from potentially harmful blood-borne compounds from elsewhere in the body. Depression has been linked to increased BBB permeability [78] and studies of CSF markers in depression suggest that the increase may reverse with successful treatment [79]. It was suggested that VEGF may play a role in this increased permeability [80]. In a mouse model of depression, claudin 5 (a tight cell junction protein) was decreased in the nucleus accumbens. In two small human studies from the same group, claudin 5 gene expression was downregulated in the nucleus accumbens in patients with depression [81, 82]. Our findings again contradict these previous studies as we found no evidence of a relationship between claudin 5, occludin or JAM-A levels and depression, at least in the brain areas under investigation, suggesting that BBB in these brain areas does not become more leaky.

Strengths of our study include the clear diagnoses in the depression groups, reflecting the unique psychological autopsy approach of the Douglas Bell Brain Bank. The individuals included in the depression groups were severely affected and had all died by suicide. We also measured a wide range of markers of vascular changes, which increase the confidence in our findings. We quantified Aβ by both ELISA and immunohistochemistry (the current gold standard), and in both the OFC and the DLPFC, again increasing the confidence in our findings. To exclude any effects of synucleinopathies, we also measured α-synuclein, a potential confounder in some of the previous studies.

Weaknesses include the relatively small numbers in the early-life depression and early-life control groups, a reflection of the scarcity of post-mortem brain tissue from this age group. Whilst the sample size in this study is relatively small, raising the possibility of selection bias, the Douglas Bell Brain Bank collects tissue from all individuals who have died by suicide in the Quebec region whose relatives give consent. We obtained tissue from as many individuals as possible for this study and it is known that human post-mortem tissue is a scarce resource. Individuals in the depression groups had to meet DSM criteria for depression in the 2 years prior to their demise, which could have meant that not all individuals met depression criteria at the time of their demise. As all of the individuals in the depression groups died by suicide, this is however much less likely. It is also possible that some individuals in the later-life depression group may have been suffering from MCI, but we made every effort to exclude individuals with cognitive impairment from this group. It is possible that some individuals in the early AD group had undiagnosed depression but again we made all possible efforts to exclude individuals with depression from the early AD group. It is also possible that some individuals with undiagnosed depression or sub threshold depressive symptoms could have been included in the control groups, but the psychological autopsy performed by the Douglas Bell Brain Bank, we feel, reduced the risk of this occurring. Likewise, it was not possible to obtain information on previous psychiatric history from the individuals in the depression groups and their control groups. Finally, because all of the individuals with depression in this study died by suicide, this may reduce the generalisability of our findings. Due to a lack of fresh frozen tissue, in the medial temporal lobe, we assessed Aβ and tau by immunohistochemistry only, but as previously mentioned immunohistochemistry is the current gold standard for measuring these proteins.

In conclusion, we report that a relatively small cohort of individuals with later-life depression had no evidence that of increased markers of AD- or Lewy body-type neurodegeneration, or cerebral hypoperfusion or increased BBB leakiness in the brain areas under investigation. The findings of our study suggest that later-life depression might rather be a risk factor for the later development of AD than a consequence of early neurodegeneration change. Future studies should assess the mechanisms by which depression may be associated with an increased risk of AD.

### Supplementary Information

**Additional file 1: Figure S1.** The ICAM-1: Collagen IV ratio in the DLPFC. The evidence of blood-brain barrier leakiness in the DLPFC was driven a single high outlier value, and the absence of observable ICAM-1 labelling in many cases. Mann-Whitney test (U=62, *p*=0.0254).

## Data Availability

As this dataset contains potentially identifiable clinical information on rare events (suicide), it has not been made publicly available. Phenotypic information on individuals who donated tissue to the Douglas Bell brain bank is available on request by bona fide researchers.
