# Correction: Is later‑life depression a risk factor for Alzheimer’s disease or a prodromal symptom: a study using post‑mortem human brain tissue?

**DOI:** 10.1186/s13195-024-01404-z

**Published:** 2024-02-13

**Authors:** Lindsey I. Sinclair, Asher Mohr, Mizuki Morisaki, Martin Edmondson, Selina Chan, A. Bone‑Connaughton, Gustavo Turecki, Seth Love

**Affiliations:** 1 Dementia Research Group, Faculty of Health Sciences, University of Bristol, Southmead Hospital, Level 1 Learning & Research Building, Bristol, BS10 5NB UK; 2 Douglas Institute, Department of Psychiatry, McGill University, Montreal, Canada; 3 Department of Life Sciences, Warwick University, Warwick, UK

**Correction: Alz Res Therapy 15, 153 (2023)**

**https://doi.org/10.1186/s13195-023-01299-2**

Following the publication of the original article [1], the authors added the funder grant number (**204813/Z/16/Z**) of the Elizabeth-Blackwell institute for Health Research University of Bristol and the Wellcome Trust Institutional Strategic Support in Competing interests section.

The original article [1] has been updated.
