# Supplementary material for: Is later-life depression a risk factor for Alzheimer’s disease or a prodromal symptom: a study using post-mortem human brain tissue?
Source: Alzheimers Res Ther. 2023 Sep 12;15:153. doi: 10.1186/s13195-023-01299-2 (PMC10496415; doi:10.1186/s13195-023-01299-2)
Supplement: Supplementary file 1 — Additional file 1: Figure S1. The ICAM-1: Collagen IV ratio in the DLPFC. The evidence of blood-brain barrier leakiness in the DLPFC was driven a single high outlier value, and the absence of observable ICAM-1 labelling in many cases. Mann-Whitney test (U=62, p=0.0254). [file 13195_2023_1299_MOESM1_ESM.docx]

**Supplementary Information**

Figure S1: The ICAM-1: Collagen IV ratio in the DLPFC. The evidence of blood-brain barrier leakiness in the DLPFC was driven a single high outlier value, and the absence of observable ICAM-1 labelling in many cases. Mann-Whitney test (U=62, p=0.0254)
